# Cook–Swartz Doppler Probe Surveillance for Free Flaps—Defining Pros and Cons

**DOI:** 10.1055/s-0040-1702922

**Published:** 2020-03-03

**Authors:** Felix J. Paprottka, Dalius Klimas, Nicco Krezdorn, Dominik Schlarb, Alexander E. J. Trevatt, Detlev Hebebrand

**Affiliations:** 1 Department of Plastic, Aesthetic, Reconstructive and Hand Surgery, Agaplesion Diakonieklinikum Rotenburg, Rotenburg (Wümme), Germany; 2 Division of Plastic Surgery, Department of Surgery, Brigham and Women's Hospital, Harvard Medical School, Boston, Massachusetts; 3 Department of Plastic and Reconstructive Surgery, Fachklinik Hornheide, Münster, Germany; 4 Department of Plastic and Reconstructive Surgery, Royal Free Hospital, London, United Kingdom

**Keywords:** flap surveillance, flap survival rate, perfusion monitoring, DIEP flap, ALT flap, TMG flap

## Abstract

**Introduction** The main postoperative complication of free flaps is perfusion compromise. Urgent intervention is critical to increase the chances of flap survival. Invasive flap perfusion monitoring with direct blood flow feedback through the Cook–Swartz Doppler probe could enable earlier detection of perfusion complications.

**Materials and Methods** Between 2012 and 2016, 35 patients underwent breast reconstruction or defect coverage after trauma with a deep inferior epigastric perforator, anterolateral thigh, transverse musculocutaneous gracilis, gracilis, or latissimus dorsi flap in our department. All flaps were monitored with a Cook–Swartz probe for 10 days postoperatively. The 20 MHz probe was placed around the arterial–venous anastomosis. A flap monitoring protocol was established for standardized surveillance of postoperative perfusion. In the event of probe signal loss, immediate surgical revision was initiated.

**Results** Signal loss was detected in 8 of the 35 cases. On return to the operating room, six were found to be true positives (relevant disruption of flap perfusion) and two were false positives (due to Doppler probe displacement). There were also two false negatives, resulting in a slowly progressive partial flap loss. Flap perfusion was restored in three of the six cases (50%) identified by the probe. Following surgical intervention, three of the six cases had persistent problems with perfusion, resulting in two total flap losses and one partial flap necrosis leading to an overall 5.7% total flap loss.

**Conclusion** Postoperative flap perfusion surveillance is a complex matter. Surgical experience is often helpful but not always reliable. The costs, false-positive, and false-negative rates associated with invasive perfusion monitoring with Cook–Swartz probe make it most appropriate for buried flaps.

**Level of Evidence** This is an original work.

Free flaps have a recognized role in defect coverage after wide resection of tumors or trauma. However, free flap transfer has the potential for postoperative complications, mostly attributable to perfusion incidents. Current literature 1 2 suggests that free flap loss after major complications is less than 5% and mainly due to arterial or venous thrombosis. 2 Immediate intervention is fundamental to flap survival. Various commercial products are available for flap perfusion monitoring; however, clinical monitoring remains the gold standard. 2 Other techniques available for monitoring include pulse oximetry, near-infrared spectroscopy, perfusion photoplethysmography, surface temperature measurement, fluorometry, microdialysis, ultrasound imaging, handheld Doppler ultrasound probe, Cook–Swartz Doppler probe (implantable), laser Doppler flowmetry, impedance plethysmography, confocal microscopy, nuclear medicine, subcutaneous pH measurement, hydrogen clearance, white light spectrometry, multispectral spatial frequency domain imaging, CO _2_ monitoring, sidestream dark field imaging, orthogonal polarized light, and hydrogen clearance. 2 3 An alternative option is to externalize a skin island of a buried flap to facilitate flap monitoring. Current literature does not suggest that any one technique leads to higher rates of flap survival compared with clinical monitoring alone. 2

This study assesses the use of the Cook–Swartz Doppler probe, which has been proposed for free flap surveillance by several authors. 4

## Materials and Methods

This retrospective study reviewed 35 free flap transfers surveyed by Cook–Swartz Doppler probe over 4 years (2012–2016). Doppler probe monitoring was indicated where reconstructions were performed using a free deep inferior epigastric perforator (DIEP), anterolateral thigh (ALT), gracilis, transverse musculocutaneous gracilis (TMG), or latissimus dorsi (LD) flap. Free flap reconstructions were performed in a single experienced microsurgery center to provide coverage to defects following trauma or cancer resections that were not amenable to simpler forms of reconstruction. Data were collected retrospectively from surgical reports, patient documentation, and free flap surveillance protocols using the hospital information system.

Intraoperatively, the Cook–Swartz Doppler probe was positioned 1 to 2 cm proximal to the venous anastomosis, avoiding the microanastomosis itself. The probe used was a crystal diode measuring ∼1 mm in diameter, which was fixed to a Silastic cuff to stabilize it in apposition to the vessel. The cuff was wrapped around the vessel and the two ends were secured using a single micro-hemoclip. The tension of the silicone cuff is important, as a tight cuff may cause venous outflow obstruction, while a loose cuff may dislodge resulting in a false positive. 5

To assess blood flow, 20 MHz sound waves are transmitted by the device and are reflected back by erythrocytes. 4 The echo is detected by the Doppler probe and transduced into an audio and visual signals. 4 Veins create a whooshing noise, whereas arteries create a lashing noise, which are received by the output device. The probe was left in situ for a minimum of 1 week during the monitoring period and was thereafter removed. 2

If signal was lost, immediate surgical intervention was initiated. Additional flap perfusion monitoring was performed by using a handheld Doppler. Clinical assessment of the flap was undertaken by looking at the color, temperature, capillary refill, bleeding, and general flap appearance. 2 A total flap loss was defined as a loss of more than 50% of the flap. Partial flap loss was defined as flap loss of less than 50%.

In the event of flap compromise, the surgeon on call was immediately informed and a single surgical revision was performed if perfusion disruption was suspected. There were no delays in returning to the operating room once the decision for surgical revision had been made. 2

### Statistical Analysis

A true positive was defined as a loss of Doppler signal indicating free flap compromise that was confirmed by surgical exploration. A false positive was defined as a loss of Doppler signal where exploration showed a well-perfused flap. A true negative was the presence of a Doppler signal with a viable flap. A false negative was the presence of a signal despite a compromised flap. 6

**Fig. 1:**
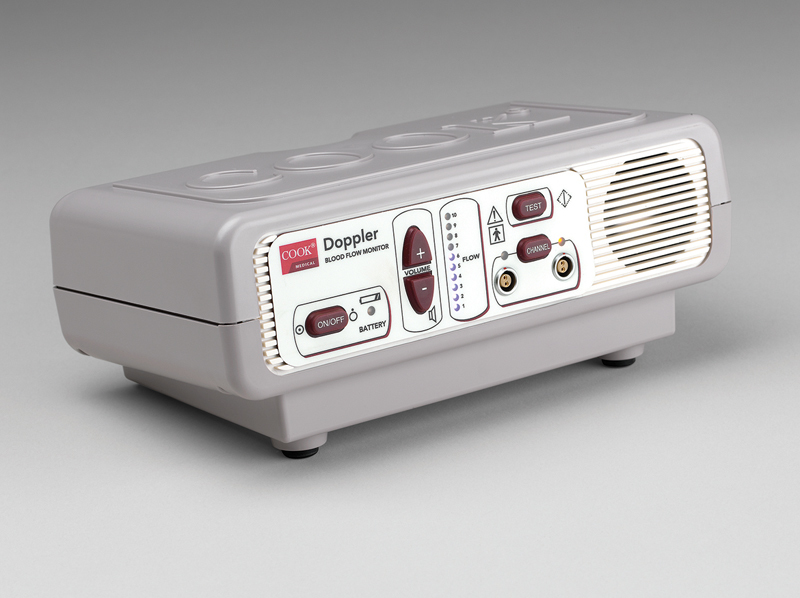
**Doppler blood flow monitor.** Cook medical Doppler blood flow monitor. Provides primary audible and secondary visual feedback of blood flow when connected to Cook–Swartz Doppler Probe. Scale from 1 to 10—visualization of audio signal. Permission for use granted by Cook Medical, Bloomington, IN.

The flap salvage rate was calculated by dividing all flaps with pedicle compromise that ultimately survived with the total number of compromised flaps. The two primary outcome measures were flap salvage rate and false-positive rate, as these were considered true tests of the efficacy when compared with just clinical monitoring. 5 Data collection and primary statistical analysis were performed using Microsoft Excel (Microsoft Corporation, Redmond, WA) ( Figs. 1 2 3 ).

**Fig. 2:**
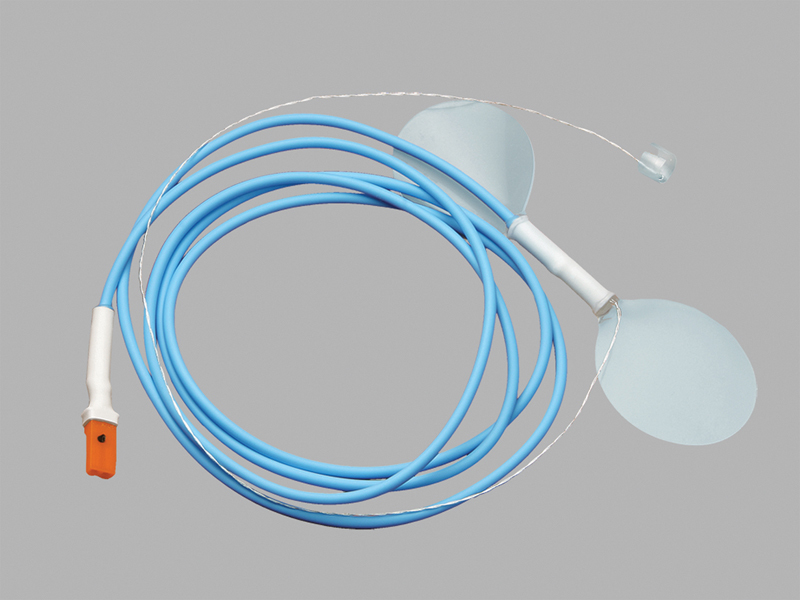
**Cook–Swartz Doppler probe.** Single-use Cook–Swartz Doppler probe. Top right corner—implantable silicone cuff with attached 20 MHz crystal, which allows monitoring of microvascular anastomoses. Permission for use granted by Cook Medical, Bloomington, IN.

**Fig. 3:**
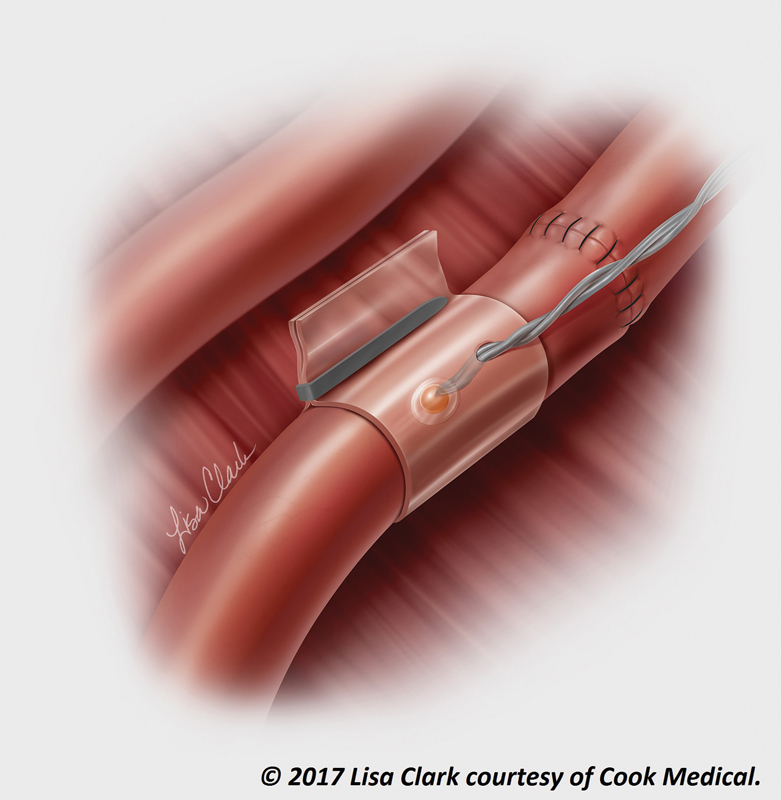
**Cook–Swartz Doppler probe.** Illustration of anastomosis with Cook–Swartz Doppler probe. Probe is usually placed on venous anastomosis. (© 2017 Lisa Clark courtesy of Cook Medical)

## Results

Between 2012 and 2016, 35 patients, who received free flap transfers using the implantable Cook–Swartz Doppler probe as the monitoring tool, were included into our study. Twenty patients were female (57%) and 15 were male (43%). Five different types of free flaps were used for reconstruction: DIEP flap, gracilis flap, TMG flap, ALT flap, and LD flap ( Table 1 ). Median surgical time was 5 hours and 52 minutes ( Table 1 ).

**Table 1:** Types of free flaps used, gender distribution, and surgical time

Flaps	DIEP	Gracilis	TMG	ALT	LD	Total
Number of patients	15 (42.9%)	3 (8.6%)	1 (2.9%)	6 (17.1%)	10 (28.6%)	35
Gender distribution	f = 15	m = 2; f = 1	m = 1	m = 5; f = 1	m = 7; f = 3	m = 15; f = 20
Median surgical time	6:20 h (4:12–14:35 h)	5:13 h (4:34–9:23 h)	4:49 h	5:54 h (4:05–11:27 h)	5:27 h (03:42–9:37 h)	5:52 h (3:42–14:35 h)

Abbreviations: ALT, anterolateral thigh; DIEP, deep inferior epigastric perforator; f, female; LD, latissimus dorsi; m, male; TMG, transverse musculocutaneous gracilis.

In 25 patients, no perfusion complications were detected. Ten patients had perfusion disturbances noted during the postoperative surveillance period. Signal losses were detected in 8 out of 35 cases. Immediate surgical revision showed six true positives (relevant disturbances of flap perfusion) and two false positives (two dislocations of Doppler probe). During surgical revision, thrombectomy and reanastomosis were performed if required and flap perfusion was restored in three out of six (50%) cases. In the three cases where perfusion could not be restored, two total flap losses and one partial flap necrosis occurred (5.7% total flap loss). There were two false negatives resulting in slowly progressing partial flap loss, resulting in flap salvage rate of 37.5%. Doppler probe results are presented in Table 2 .

**Table 2:** Cook–Swartz Doppler probe results

Doppler test	Pedicle compromise		No pedicle compromise	
Positive	True positive	6	False positive	2
Negative	False negative	2	True negative	25

*Note* : Cook–Swartz Doppler test results compared with pedicle perfusion status in 35 patients where the device was used ( *n* = 35).

Cook–Swartz Doppler probe diagnostic test evaluation is shown in Table 3 . Cook–Swartz Doppler probe diagnostic test had a sensitivity of 75% and specificity of 92.59%. A high rate of false-negative results (25%) was given; however, it showed a low false-positive rate of 7.41%. Further analysis showed positive likelihood ratio of 10.12 and negative likelihood ratio of 0.27.

**Table 3:** Cook–Swartz Doppler probe diagnostic test evaluation

Statistic	Value	95% CI
False-positive rate	7%	1–24%
False-negative rate	25%	3–65%
Sensitivity	75%	35–97%
Specificity	93%	76–99%
Positive likelihood ratio	10.12	2.52–40.75
Negative likelihood ratio	0.27	0.08–0.90

Abbreviation: CI, confidence interval.

*Note* : Calculated statistical values according to Cook–Swartz Doppler probe diagnostic test results and the pedicle blood flow status. False-positive and false-negative rates, sensitivity, specificity, and positive and negative likelihood ratios were assessed.

## Discussion

There is universal agreement that early intervention is key to successful flap salvage following arterial or venous thrombosis, external compression, or kinking of the pedicle after free flap reconstruction. 5 Expert opinion and available scientific studies indicate that early detection of flap compromise and subsequent surgical re-exploration significantly increases the flap survival rate. 3 Clinical monitoring alone is not always sufficient to detect perfusion problems. There are several methods to indirectly monitor perfusion, oxygenation, or ischemia which vary in reliability. The Cook–Swartz Doppler probe allows the direct measurement of blood flow since a monitor is placed onto the pedicle. It can therefore help detect flap compromise before clinical ischemia becomes evident. 7

The first attempts to utilize an implantable Doppler probe for free flap monitoring were by Parker et al 8 in 1984. The Cook–Swartz probe was subsequently developed specifically for the use in free flap surgery (1988). 9 The manufacturer states that it is a reliable technique with high sensitivity due to permanent intra- and postoperative perfusion monitoring. Opinions differ about the choice of vessel to be monitored. It was initially used on the arterial pedicle, but most studies describe it to be more sensitive when placed on the venous anastomosis. 6 10 However, there are disadvantages such as the invasiveness of the technique. In addition, kinking of vessels due to probe dislocation has been reported and there can be difficulties applying the probe to small vessels.

There are a large variety of products available for flap perfusion monitoring, and limited evidence that any particular technique leads to increased rates of flap salvage in comparison to clinical monitoring alone. 2 According to Chae et al, 3 the optimal test for monitoring efficacy is the salvage rate, defined as the ability of a monitoring technique to allow early intervention and salvage of a truly compromised flap. Monitoring should be both highly specific and sensitive, measured by the rate of unwarranted returns to the operating room. 3 In this setting, we achieved a low false-positive rate of 7.41%, which could be improved with better probe fixation. However, we did expect higher sensitivity than achieved in our study (75%). We also accept the limitations of our false-negative rate, since this calculation is dependent on subjective surgical decision making. 5 In addition, positive likelihood ratio of 10.12 showed that the test is useful to rule in a flap perfusion disturbances. Although a negative Cook–Swartz Doppler probe result should not be used to rule out the disease because of low sensitivity (75%) and high negative likelihood ratio (0.27), clinical signs of bad flap perfusion are presented.

Also, the optical signal given by the device is only a plain visualization of audio signal—giving no further information about signal quality.

Opinions on the most effective form of monitoring differ in the literature. A multicenter comparison by Whitaker et al showed no difference between clinical monitoring, Cook–Swartz implantable Doppler, and microdialysis. 2 However, the majority of large cohort studies demonstrated an improvement in flap salvage rates with the Cook–Swartz probe compared with clinical monitoring alone. 1 4 5 7 11 12 Furthermore, a meta-analysis confirmed a statistically significant benefit of the Cook–Swartz probe over clinical monitoring. 5 However, none of these studies has reached the level of evidence necessary to trigger widespread uptake of the Cook–Swartz probe. 3

A systematic review performed by Chae et al (2015) stated that only implanted Doppler probes, near-infrared spectroscopy, laser Doppler flowmetry, quantitative fluorimetry, and digital photography assessment using smartphones have shown an improved flap salvage rate. 3 Of these, the Doppler probe has the largest number of comparative studies to demonstrate its effectiveness compared with clinical monitoring. 3

Our primary focus was on increasing free flap survival rate with the use of the Cook–Swartz Doppler probe. Unfortunately, however, in this study, our flap loss rate of 5.7% with the Cook–Swartz Doppler probe did not improve on the rate stated in the literature of around 2% (0–6%). 1 2

High false-positive rate has been described by several authors, 13 14 15 leading to unnecessary returns to the operating room, and therefore, potentially increasing morbidity and the treatment costs. 6 This was reiterated by Whitaker et al who showed that the use of microdialysis and the implantable Doppler significantly increases the rate of unnecessary take backs to operating room (the false-positive rate) over the use of clinical monitoring alone. 2 Some other studies state the opposite. 16 In our experience, we had 7.41% false-positive rate due to dislocation of the probe which led to unnecessary revisions and increased cost of the treatment.

There are also financial considerations: Doppler probes are relatively expensive (300 British Pounds per piece) and the output device must be purchased separately (single investment of 2,000 British Pounds), making this a significant investment. 17

A systematic review performed by Poder and Fortier claimed that, because of increase in the flap salvage rate compared with standard clinical monitoring, the use of an implantable Doppler could avoid the need for new free flap surgery in two out of every 100 patients, and up to four or five cases per 100 patients for buried flaps. 18 Avoiding new surgery could partially compensate the additional cost of the Doppler probe or even reduce the overall cost depending on the initial flap salvage rate and the type of free flap (buried vs. non-buried).

In certain cases, such as monitoring of buried free muscle flaps or surveillance of arteriovenous loops during preparation for free flaps, the need for an implanted Doppler probe becomes more apparent. Until the cost of implanted Doppler probes is reduced, their widespread use is likely to be limited to special cases such as these.

## Conclusion

Postoperative flap perfusion surveillance remains a complex and difficult matter. Free flap perfusion complications are often identified too late, which reduces flap salvage rate and leads to increased total flap loss rate. Surgical experience is often helpful but not always reliable at identifying a compromised flap. The Cook–Swartz implantable Doppler system is a simple, safe, and valid option for continuous real-time free flap monitoring, and is a useful adjunct to clinical monitoring. However, fixation of the Doppler probe needs to be improved to reduce false-positive rate resulting from probe dislocation and unnecessary revisions. Our experience also suggests that the benefit–cost ratio needs to be more balanced. More comprehensive studies would be useful to determine efficacy of Doppler probe monitoring, particularly in comparison to other monitoring devices.

## References

[1] Wax M K The role of the implantable Doppler probe in free flap surgery Laryngoscope 2014 124 01 S1 S12 10.1002/lary.24569 24375425

[2] Whitaker I S Rozen W M Chubb D Postoperative monitoring of free flaps in autologous breast reconstruction: a multicenter comparison of 398 flaps using clinical monitoring, microdialysis, and the implantable Doppler probe J Reconstr Microsurg 2010 26 06 409 416 20221988 10.1055/s-0030-1249607

[3] Chae M P Rozen W M Whitaker I S Current evidence for postoperative monitoring of microvascular free flaps: a systematic review Ann Plast Surg 2015 74 05 621 632 23038130 10.1097/SAP.0b013e3181f8cb32

[4] de la Torre J Hedden W Grant J H III Gardner P M Fix R J Vásconez L O Retrospective review of the internal Doppler probe for intra- and postoperative microvascular surveillance J Reconstr Microsurg 2003 19 05 287 290 14506574 10.1055/s-2003-42495

[5] Rozen W M Chubb D Whitaker I S Acosta R The efficacy of postoperative monitoring: a single surgeon comparison of clinical monitoring and the implantable Doppler probe in 547 consecutive free flaps Microsurgery 2010 30 02 105 110 19790183 10.1002/micr.20706

[6] Ho M W Cassidy C Brown J S Shaw R J Bekiroglu F Rogers S N Rationale for the use of the implantable Doppler probe based on 7 years' experience Br J Oral Maxillofac Surg 2014 52 06 530 534 24721166 10.1016/j.bjoms.2014.03.014

[7] Rozen W M Enajat M Whitaker I S Lindkvist U Audolfsson T Acosta R Postoperative monitoring of lower limb free flaps with the Cook–Swartz implantable Doppler probe: a clinical trial Microsurgery 2010 30 05 354 360 19967762 10.1002/micr.20720

[8] Parker P M Fischer J C Shaw W W Implantable pulsed Doppler cuff for long-term monitoring of free flaps: a preliminary study Microsurgery 1984 5 03 130 135 6493028 10.1002/micr.1920050307

[9] Swartz W M Jones N F Cherup L Klein A Direct monitoring of microvascular anastomoses with the 20-MHz ultrasonic Doppler probe: an experimental and clinical study Plast Reconstr Surg 1988 81 02 149 161 3336646 10.1097/00006534-198802000-00001

[10] Swartz W M Izquierdo R Miller M J Implantable venous Doppler microvascular monitoring: laboratory investigation and clinical results Plast Reconstr Surg 1994 93 01 152 163 8278470 10.1097/00006534-199401000-00024

[11] Paydar K Z Hansen S L Chang D S Hoffman W Y Leon P Implantable venous Doppler monitoring in head and neck free flap reconstruction increases the salvage rate Plast Reconstr Surg 2010 125 04 1129 1134 20335864 10.1097/PRS.0b013e3181d0ab23

[12] Guillemaud J P Seikaly H Cote D Allen H Harris J R The implantable Cook–Swartz Doppler probe for postoperative monitoring in head and neck free flap reconstruction Arch Otolaryngol Head Neck Surg 2008 134 07 729 734 18645123 10.1001/archotol.134.7.729

[13] Lineaweaver W C The implantable Doppler probe Plast Reconstr Surg 1988 82 06 1099 1100 3059384 10.1097/00006534-198812000-00038

[14] Lineaweaver W Techniques of monitoring buried fasciocutaneous free flaps Plast Reconstr Surg 2009 124 05 1729 1731 20009871 10.1097/PRS.0b013e3181b98d34

[15] Rosenberg J J Fornage B D Chevray P M Monitoring buried free flaps: limitations of the implantable Doppler and use of color duplex sonography as a confirmatory test Plast Reconstr Surg 2006 118 01 109 113, discussion 114–115 16816680 10.1097/01.prs.0000221113.78244.8c

[16] Smit J M Whitaker I S Liss A G Audolfsson T Kildal M Acosta R Post operative monitoring of microvascular breast reconstructions using the implantable Cook–Swartz Doppler system: a study of 145 probes & technical discussion J Plast Reconstr Aesthet Surg 2009 62 10 1286 1292 18675608 10.1016/j.bjps.2008.06.007

[17] Schmulder A Gur E Zaretski A Eight-year experience of the Cook–Swartz Doppler in free-flap operations: microsurgical and reexploration results with regard to a wide spectrum of surgeries Microsurgery 2011 31 01 1 6 20683856 10.1002/micr.20816

[18] Poder T G Fortier P H Implantable Doppler in monitoring free flaps: a cost-effectiveness analysis based on a systematic review of the literature Eur Ann Otorhinolaryngol Head Neck Dis 2013 130 02 79 85 23182889 10.1016/j.anorl.2012.07.003

